# Impact of the COVID-19 Pandemic on Iron Overload Assessment by MRI in Patients with Hemoglobinopathies: The E-MIOT Network Experience

**DOI:** 10.3390/tomography9050136

**Published:** 2023-09-11

**Authors:** Antonella Meloni, Laura Pistoia, Amalia Lupi, Riccardo Righi, Antonino Vallone, Massimiliano Missere, Stefania Renne, Priscilla Fina, Ada Riva, Maria Rita Gamberini, Valerio Cecinati, Francesco Sorrentino, Rosamaria Rosso, Giuseppe Messina, Paolo Ricchi, Vincenzo Positano, Sophie Mavrogeni, Emilio Quaia, Filippo Cademartiri, Alessia Pepe

**Affiliations:** 1 Department of Radiology, Fondazione G. Monasterio CNR-Regione Toscana, 56124 Pisa, Italy; antonella.meloni@ftgm.it (A.M.); laura.pistoia@ftgm.it (L.P.); positano@ftgm.it (V.P.); fcademartiri@ftgm.it (F.C.); 2 Unità Operativa Complessa Bioingegneria, Fondazione G. Monasterio CNR-Regione Toscana, 56124 Pisa, Italy; 3 Istituto di Radiologia, Dipartimento di Medicina, Università di Padova, 35128 Padova, Italy; amalia.lupi@phd.unipd.it (A.L.); emilio.quaia@unipd.it (E.Q.); 4 Diagnostica per Immagini e Radiologia Interventistica, Ospedale del Delta, 44023 Lagosanto, Italy; riccardo.righi@ausl.fe.it; 5 Reparto di Radiologia, Azienda Ospedaliera “Garibaldi” Presidio Ospedaliero Nesima, 95126 Catania, Italy; ninovallone@hotmail.com; 6 Unità Operativa Complessa Radiodiagnostica, Gemelli Molise SpA, Fondazione di Ricerca e Cura “Giovanni Paolo II”, 86100 Campobasso, Italy; massimiliano.missere@gmail.com; 7 Struttura Complessa di Cardioradiologia, Presidio Ospedaliero “Giovanni Paolo II”, 88046 Lamezia Terme, Italy; stefania.renne@virgilio.it; 8 Unità Operativa Complessa Diagnostica per Immagini, Ospedale “Sandro Pertini”, 00157 Roma, Italy; priscilla.fina@gmail.com; 9 Struttura Complessa di Radiologia, Ospedale “SS. Annunziata” ASL Taranto, 74121 Taranto, Italy; ada.riva@yahoo.it; 10 Unità Operativa di Day Hospital della Talassemia e delle Emoglobinopatie, Dipartimento della Riproduzione e dell’Accrescimento, Azienda Ospedaliero-Universitaria “S. Anna”, 44124 Cona, Italy; gamberinimariarita@gmail.com; 11 Struttura Semplice di Microcitemia, Ospedale “SS. Annunziata” ASL Taranto, 74100 Taranto, Italy; valerio.cecinati@asl.taranto.it; 12 Unità Operativa Semplice Dipartimentale Day Hospital Talassemici, Ospedale “Sant’Eugenio”, 00143 Roma, Italy; francesco.sorrentino@aslroma2.it; 13 Unità Operativa Talassemie ed Emoglobinopatie, Azienda Ospedaliero-Universitaria Policlinico “Vittorio Emanuele”, 95100 Catania, Italy; rosellinarosso@gmail.com; 14 Centro Microcitemie, Grande Ospedale Metropolitano “Bianchi-Melacrino-Morelli”, 89100 Reggio Calabria, Italy; gspmessina@virgilio.it; 15 Unità Operativa Semplice Dipartimentale Malattie Rare del Globulo Rosso, Azienda Ospedaliera di Rilievo Nazionale “A. Cardarelli”, 80131 Napoli, Italy; paolo.ricchi@aocardarelli.it; 16 Onassis Cardiac Surgery Center, 17674 Athens, Greece; sophie.mavrogeni@gmail.com

**Keywords:** COVID-19, iron overload, magnetic resonance imaging

## Abstract

Background. The E-MIOT (Extension-Myocardial Iron Overload in Thalassemia) project is an Italian Network assuring high-quality quantification of tissue iron overload by magnetic resonance imaging (MRI). We evaluated the impact of the COVID-19 pandemic on E-MIOT services. Methods. The activity of the E-MIOT Network MRI centers in the year 2020 was compared with that of 2019. A survey evaluated whether the availability of MRI slots for patients with hemoglobinopathies was reduced and why. Results. The total number of MRI scans was 656 in 2019 and 350 in 2020, with an overall decline of 46.4% (first MRI: 71.7%, follow-up MRI: 36.9%), a marked decline (86.9%) in the period March–June 2020, and a reduction in the gap between the two years in the period July–September. A new drop (41.4%) was recorded in the period October–December for two centers, due to the general reduction in the total amount of MRIs/day for sanitization procedures. In some centers, patients refused MRI scans for fear of getting COVID. Drops in the MRI services >80% were found for patients coming from a region without an active MRI site. Conclusions. The COVID-19 pandemic had a strong negative impact on MRI multi-organ iron quantification, with a worsening in the management of patients with hemoglobinopathies.

## 1. Introduction

Thalassemia and sickle cell disease are rare inherited red blood cell disorders. Thalassemias stem from quantitative irregularities in the production of hemoglobin’s globin chain [1], with thalassemia major being the most severe form. Conversely, sickle cell disease arises from qualitative anomalies in the structure of β-globin chains [2]. Both diseases are associated with a remarkable burden and are characterized by multisystem involvement and the need for intensive life-long therapy and follow-up [3]. Repeated blood transfusions, besides being mandatory for the survival of patients with thalassemia major [4], are also frequently adopted in patients with thalassemia intermedia and sickle cell disease to prevent or treat specific complications [5,6,7]. Due to the absence of efficient mechanisms for expelling surplus iron from the body, iron overload in the vital organs of the body is a dreaded and unavoidable consequence of ongoing transfusion therapy treatment [8]. Moreover, in patients with thalassemia iron overload can also occur in the absence of transfusions, due to increased intestinal iron absorption [9]. Due to the high toxicity, iron overload can lead to unfavorable clinical consequences which include hepatic fibrosis, cirrhosis, and increased hepatocellular carcinoma risk due to hepatic iron overload [10,11], hypothyroidism, hypoparathyroidism, growth retardation, hypogonadism, and diabetes mellitus determined by iron overload in endocrine organs [12,13,14,15,16], and heart failure (HF) due to myocardial iron overload [17,18]. In thalassemia, the causal relation between iron overload and mortality has been clearly demonstrated [19,20] and in sickle cell disease, iron burden can cause up to 11% of deaths [21]. Iron chelation therapy is the mainstay of prevention and reversal of tissue iron overload. Nowadays the primary goal of chelation therapy has become the permanent maintenance of safe levels of body iron, achieved by tailoring the chelator types and dosage according to the individual patient’s needs. Thanks to its ability of noninvasively quantify tissue iron overload, the T2* magnetic resonance imaging (MRI) technique has gained a unique role in evaluating the efficacy of the iron chelation therapy and has revolutionized the management of patients with hemoglobinopathies, with a significant impact on their prognosis [19,20,22]. In Italy, the E-MIOT (Extension—Myocardial Iron Overload in Thalassemia) Network is a collaborative project among radiological MRI centers and clinical centers involved in the care of patients with rare hemoglobinopathies (hematological, pediatric, internal medicine, transfusional services) that has assured high-quality quantification of iron in vital organs such as heart, liver, and pancreas for a significant number of patients [16,20].

Italy was one of the first Western countries hit by the Coronavirus disease 2019 (COVID-19) pandemic, caused by the novel severe acute respiratory syndrome coronavirus 2 (SARS-CoV-2). To limit the spread of the virus, the entire country was locked down on 9 March 2020, with a national quarantine which severely restricted the movement of the population except for necessity, work, and health circumstances and imposed a significant social distancing. Moreover, since then the National Health System has been restructured to cope with the increased demand for hospital and intensive care unit beds for patients infected with COVID-19 and to reduce the risk of cross-infection. So, to face the increased pressure and to minimize patient contact with healthcare professionals, the access to care for patients without COVID-19-related disorders has been strongly reduced through the interruption of non-emergency admissions and of many ambulatory services and the postponement or cancellation of some elective procedures and deferable diagnostic evaluations [23,24,25]. Besides the restrictions imposed and the postponement of not indispensable activities, another important factor in Italy as well as in many countries has contributed to the disruption of healthcare services: the patients’ mistrust and reluctance to seek medical care due to fear of contracting the virus. Particularly in Italy, the consideration of the hospitals as a potential source of infection has been strongly reinforced by the fact that in the Lombardy region the hospitals themselves effectively became the new epicenter of infections [26].

Based on this background, our multicenter study aimed to evaluate the real-world impact of the COVID-19 pandemic on MRI services for iron overload quantification in patients with rare hemoglobinopathies in Italy.

## 2. Materials and Methods

### 2.1. E-MIOT Network

The E-MIOT study complies with the Declaration of Helsinki and was approved by the institutional Ethics Committees.

**Figure 1:**
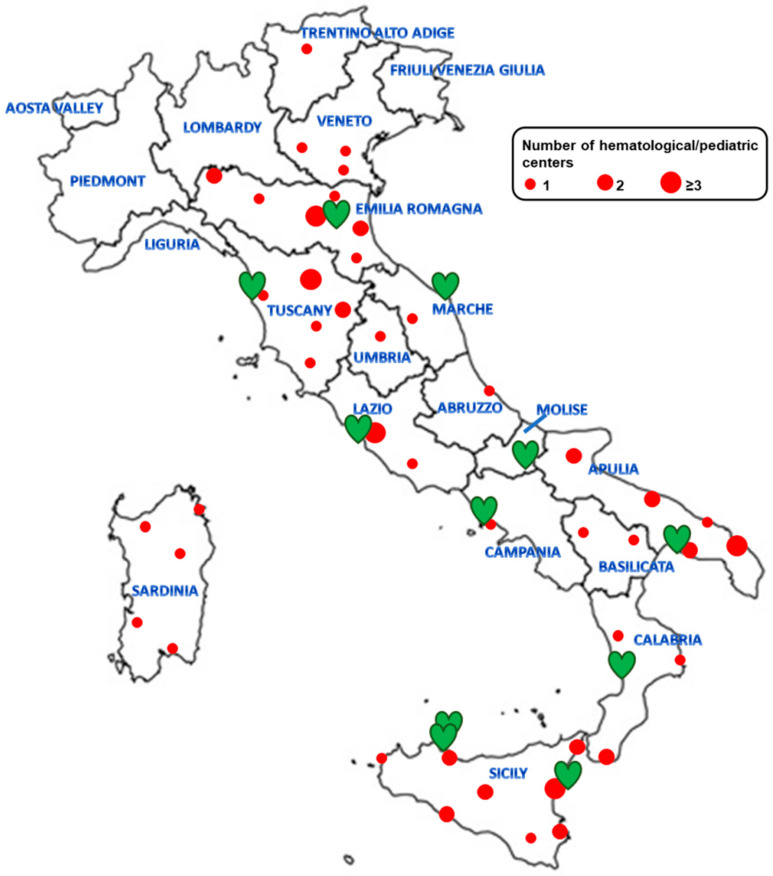
Locations of the E-MIOT clinical centers involved in the care of patients with rare hemoglobinopathies (red circles) and MRI centers (green hearts) in Italy.

E-MIOT is an Italian network comprising 66 hematological, pediatric, internal medicine, and transfusional centers and 11 MRI sites (Figure 1).

The hematological/pediatric centers enroll the patients, according to the following inclusion criteria: (1) male and female patients, of all ages, affected by thalassemia syndromes or structural hemoglobin variants and needing a MRI for iron burden quantification; (2) written informed consent to the study participation; (3) written authorization for use and disclosure of protected health information; (4) absence of absolute contraindications to MRI.

Each MRI site is equipped with one conventional clinical 1.5T scanner produced by one of three main vendors (GE Healthcare, Milwaukee, WI, USA; Philips Healthcare, Best, The Netherlands; Siemens Healthineers, Erlangen, Germany) and has joined the E-MIOT network after a validation procedure ensuring standardization of image acquisition and post-processing [27,28]. In all sites, the MRI protocol for iron overload assessment involves the acquisition of a multi-echo gradient-echo T2* sequence (10 echo times acquired in a single end-expiratory breath-hold with an echo spacing of 2.26 ms) of a mid-transverse hepatic slice [29], five or more axial slices covering the whole abdomen and containing the pancreas [30], and three parallel short-axis views (basal, medium, and apical) of the left ventricle (LV) [31,32]. T2* image analysis is performed by trained operators with a high level of expertise using custom-written, previously validated software (HIPPO MIOT^®,^, Version 2.0, Consiglio Nazionale delle Ricerche and Fondazione Toscana Gabriele Monasterio, Pisa, Italy, Year 2015) [33]. Hepatic T2* is calculated in a circular region of interest (ROI) of standard dimension defined in a homogeneous area of the hepatic parenchyma [34] and it is converted into liver iron concentration (LIC) by using the Wood’s calibration curve [35,36]. The pancreas T2* is obtained by averaging the T2* values obtained in three different pancreatic regions (head, body, and tail) [37]. The myocardial T2* distribution is mapped into a 16-segment LV model, according to the AHA/ACC model [38]. An appropriate correction map is applied to compensate for susceptibility artifacts [33]. The global heart T2* value is obtained by averaging all segmental T2* values.

The following diagnostic criteria are applied for the identification of iron overload. A MRI LIC < 3 mg/g dry weight (dw) indicates no significant hepatic iron overload [39]. The lowest threshold of normal T2* pancreatic value is 26 ms [30]. A measurement of 20 ms is taken as a “conservative” normal value for segmental and global heart T2* values [40].

The T2* results are automatically sent to the web-based E-MIOT database, created to connect the hematological and MRI centers. The users at the hematological centers register the enrolled patients and insert all the required anamnestic, laboratory, and clinical data. Importantly, they also enable the MRI centers where patients are sent for the exam to read this information.

All patient data are updated at each MRI follow-up, performed by protocol every 18 ± 3 months.

### 2.2. Data Collection

Four MRI centers were not considered in the present study, due to the following reasons. The two sites in Palermo and the site in Ancona suspended their activities in the two considered years for technical reasons, internal reorganization, or scanner replacement. The site in Napoli joined the Network and completed the validation procedure in 2020.

The coordinating center of the E-MIOT Network (Pisa) has access to all T2* data contained in the E-MIOT database and can therefore determine the total number of patients examined per center (Pisa and all peripheral sites). The activity of the MRI centers of the E-MIOT Network in the year 2020 was compared to the activity in the same months of 2019.

To evaluate if the availability of MRI slots for patients with hemoglobinopathies was reduced and the reasons, a specific survey was created in Excel by the coordinating center of the E-MIOT Network and the spreadsheet was emailed to all the MRI operators. The completed survey was emailed back to the E-MIOT coordinating center and the data collated.

### 2.3. Statistical Analysis

All data were analyzed using SPSS version 27.0 (SPSS Inc., Chicago, IL, USA) statistical package.

Continuous variables were described as mean ± standard deviation (SD) and categorical variables were expressed as frequencies and percentages.

The normality of the distribution of the parameters was assessed by using the Kolmogorov-Smirnov test.

For continuous values with normal distribution, comparisons between two groups were made by independent-samples *t*-test, while Wilcoxon’s signed rank test was applied for continuous values with non-normal distribution. The χ^2^ test was used for the comparison of non-continuous variables.

A two-tailed *p* of 0.05 was considered statistically significant.

## 3. Results

The total number of T2* MRI scans was 656 in 2019 and 350 in 2020, leading to an overall decline of 46.4%.

Table 1 shows the clinical and demographic characteristics of the patients scanned in the years 2019 and 2020. No difference between the 2019 and the 2020 cohorts was detected in terms of mean age sex, and type of hematological disease. In both cohorts, the vast majority of patients were affected by thalassemia major. In comparison with the year 2019, the drop in MRI procedure volume in 2020 was 28.6% for homozygous sickle cell disease patients, 46.7% for patients with a combination of the sickle cell mutation and beta-thalassemia mutation, 44.3% for patients with thalassemia intermedia, and 58.4% for patients with thalassemia major. The MRI examinations were divided into first (or baseline) MRI and follow-up MRI (approximately every 18 months after the first MRI). The proportion of baseline MRIs was significantly higher (*p* < 0.0001) in the year 2019 than in the year 2020 (28.0% vs. 14.9%). There was a 71.7% decline in the first MRIs and a 36.9% decline in follow-up MRIs in 2020 compared to 2019.

MRI LIC values, global pancreas T2* values, and global heart T2* values were comparable between the 2019 and 2020 patient cohorts.

**Figure 2:**
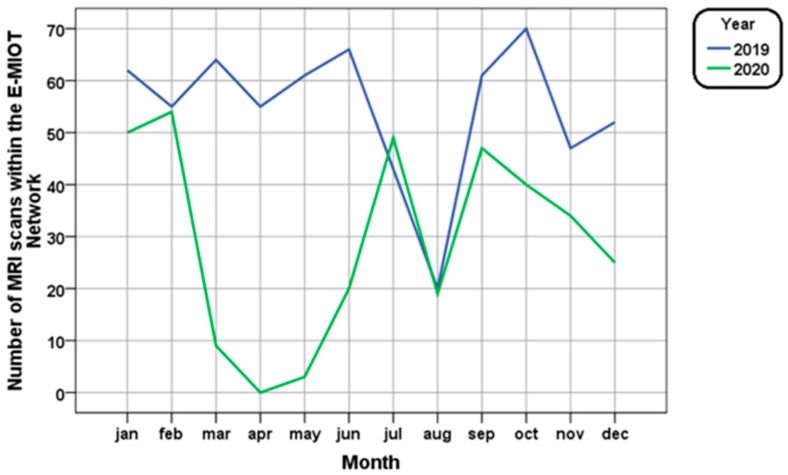
Monthly evolution in number of T2* MRI scans in the years 2019 and 2020.

The comparison month by month between the two years is shown in Figure 2. A marked decline (86.9%) in the four-month period March–June 2020 was detected. Specifically, no patient with hemoglobinopathy could undergo an MRI scan during the Italian lockdown (9 March 2020–3 May 2020) and, when directly comparing pre-COVID and COVID time in the period from 11 May to 30 June, an 81.9% decline in the number of MRIs performed in 2020 compared to 2019 was detected. There was a reduction in the gap between the two years in the three-month period July–September and a new decline (41.4%) in the three-month period October–December.

Table 2 shows the number of patients scanned in each of the seven included MRI sites. Each MRI center had a specific absorption capacity which drastically decreased in the year 2020. In both years 2019 and 2020, more than half of the total patients were examined in the centers of Ferrara and Pisa.

**Figure 3:**
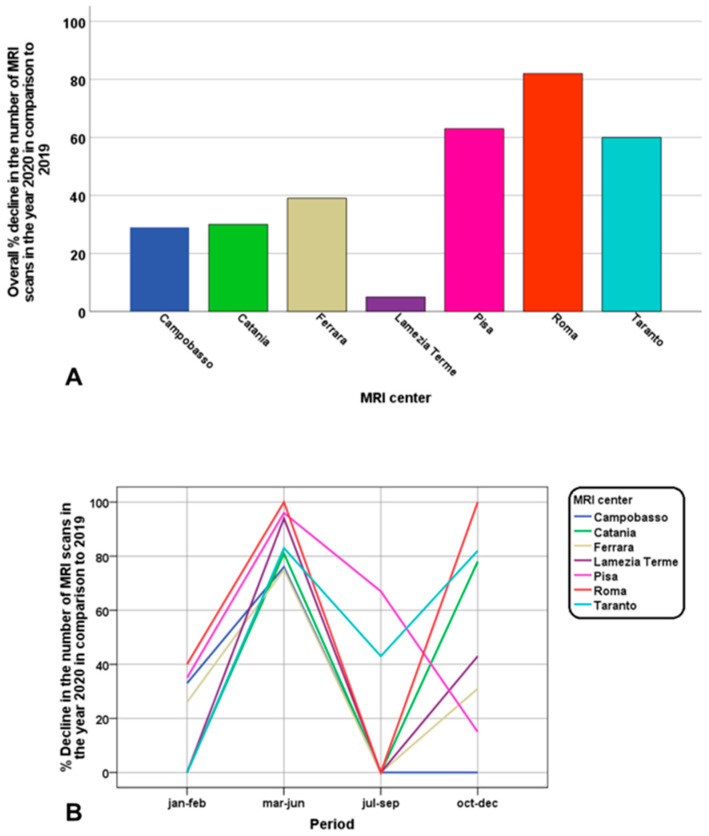
(**A**) Bar charts showing the overall percentage decline in the number of T2* MRI scans in 2020 compared to 2019 for each MRI site involved in the E-MIOT project. (**B**) Percentage decline in the number of T2* MRI scans in certain periods of 2020 compared to the same periods in 2019 for each MRI site involved in the E-MIOT project.

The overall percentage decline (year 2020 vs. 2019) in the number of T2* MRI scans for each MRI center is shown in Figure 3A. The site in Roma experienced the greatest percentage of reduction (81.5%), followed by the site in Pisa (63.1%). Figure 3B highlights the percentage decline in each defined period. If no decline or an increase were present, the vertical axis was set at 0. All centers experienced a significant drop in the number of T2* MRIs in the four-month period March–June (from 75% to 100%). In the three-month period July–September only the centers of Pisa and Taranto dropped the number of T2* MRIs (67.7% and 42.9%, respectively) due to the rescheduling of all the MRI appointments deleted during the lockdown. In the three-month period October–December a reduction of the T2* MRI scans was experienced by all centers, except for Campobasso. In the centers of Ferrara and Lamezia Terme the reduction was the consequence of the general reduction in the number of the total MRIs scheduled per day for the sanitation procedures. In the other centers, the availability for T2* MRI scans was unchanged in comparison to 2019, but the patients refused the MRI for fear of getting sick from COVID-19.

**Figure 4:**
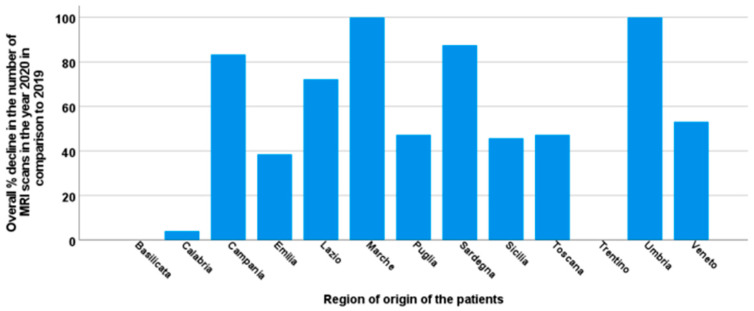
Bar charts showing the overall percentage decline in the number of T2* MRI scans in 2020 compared to 2019 considering the region of provenience of the patients.

Regarding the regions of origin of the patients, our data indicated reductions in the MRI services by > 80% for those patients coming from a region without an active MRI site (Marche, Campania, Umbria, and Sardinia) (Figure 4). There were two exceptions: the hematological center in the Trentino region, which joined the Network only in 2020, and the hematological center in the city of Matera (Basilicata), which is an hour away by car from the MRI center of Campobasso.

In 2021 there was a return to the normal activity, with a total number of T2* MRI scans of 646.

## 4. Discussion

Due to the need to reduce the chance of transmitting the virus to either patients or healthcare workers and to meet a surge in demand, the COVID-19 pandemic has drastically modified healthcare delivery, leading to the deferral of different diagnostic and therapeutic procedures [23,41]. To our knowledge, this is the first study to assess the impact of COVID-19 on the provision of MRI services for the quantification of iron overload in patients with thalassemia and sickle cell disease. The Italian experience of the E-MIOT Network showed a significant reduction (almost 50%) in the year 2020 in comparison to 2019 in the number of MRI exams performed. When considering the lockdown period, this decline was shown to be 100%. After the lockdown, the MRI services for the iron overload quantification were not immediately back on normal track and in the following month and a half a decline of > 80% in the number of patients studied in 2020 compared to 2019 was detected. All the necessary protective measures regarding COVID-19 (i.e., fewer patients per time unit) and in some cases the reorganization of the MRI services prevented an immediate rise in the number of MRI accesses for the iron overload assessment. In the three-month period July–September 2020, the numbers were steady compared to 2019 for the majority of the centers. Very probably, this return close to baseline levels was a direct consequence of the low incidence of COVID-19 during the summer of 2020. Indeed, by the autumn, with the increase in the number of cases of COVID-19 and the arrival of the second wave, there was a new decline in the number of T2* MRI scans, mainly due to the refusal of the patients, since only in two of the 7 considered centers there was a real reduction in the number of scheduled MRIs for patients with hemochromatosis. Patients with hemoglobinopathies are at increased risk of severe COVID-19 disease. The virus can attack heme and hemoglobin metabolisms, enhancing the adverse effects of the already impaired hemoglobin metabolism and accelerating the progression of severe symptoms [42]. Moreover, different underlying and frequent conditions, including severe iron overload, heart disease, liver disease, diabetes, adrenal insufficiency, complications involving the lungs and the immune system, kidney disease, splenectomy, or asplenia, strongly contribute to increased susceptibility to infection [43,44,45]. So, it is not surprising that many patients, aware of their vulnerability, preferred to postpone the MRI due to fear of being infected with the SARS-CoV-2. The majority of the patients were under regular transfusion therapy and likely they decided to frequent the hospital environment only for this procedure, considered by themselves essential and not postponable, conversely to the MRI for iron quantification. Importantly, the transfusion procedure could be performed close to home, while many patients had to move to a different city or region to perform a MRI scan. So, it is plausible that, besides the personal fear, the adopted restrictions on traveling and the highly recommended prevention measures prioritizing social distancing, such as shutting down public transportation and avoiding carpooling have strongly influenced the patient behavior. In fact, the highest decline in MRI scans has been observed for these patients living in a region without an active MRI site. The fact that the distance between the patient’s location and the MRI site location had a significant impact on the utilization of medical services like MRI scans further underlies the importance of spreading the availability of MRI scans for iron quantification close to the patients. Generally speaking, addressing geographic barriers is crucial for ensuring equitable access to healthcare services and improving patient outcomes. Finally, since Italy’s economy has been seriously impacted by the COVID-19 pandemic, the constrained resources may have contributed to limiting the capability of the patients to reach the MRI sites.

Importantly, our report demonstrated a higher decline (almost double) in the number of baseline MRIs compared to the follow-up MRIs, suggesting that, putting aside the fear, the patients who have already done a MRI are more aware of the importance of this type of exam for their management. Indeed, although it is true that for hematologists the MRI T2* has become the key for tailor-made chelation therapies customized for each patient [46], it is equally true that for the patients it represents the opportunity to directly “touch” the iron status of their vital organs, improving their compliance in the chelation therapy [47].

The downstream impacts of the COVID-19-related contraction in T2* MRI scans are presently unknown, but adverse consequences for some patients presenting with worsening of their iron levels may be inevitable. An increase in the frequency of iron-related complications may be expected in the next few years. So, on one side the clinical centers involved in the care of patients with rare hemoglobinopathies should be ready for a “rebound” effect. On the other side, it is strongly recommended to plan strategies to recover all the lost or postponed exams and to avoid further restrictions on the MRI availability in the occasion of new waves of COVID-19 cases. Future longitudinal studies should be performed to clarify the impact of this contraction on patients’ outcomes.

### Limitations

Only the MRI centers of the E-MIOT Network were considered, and it cannot be taken for granted that our results could be fully extended to other Italian MRI centers performing iron overload quantifications.

This research was conducted in only one country and more studies are needed to evaluate the impact of COVID-19 on iron overload quantifications all over the world.

The findings of the present study apply only to the period investigated, and, thanks to the vaccination campaign, different results may be found when considering the year 2021.

## 5. Conclusions

The COVID-19 pandemic had a strong negative impact on the multi-organ iron quantification by MRI, which may seriously worsen the prognosis of patients with hemoglobinopathies. Strategies to ensure proven lifesaving MRI exams and to reassure patients about the health safety of the hospitals are firmly recommended in this population where morbidity and mortality have been demonstrated strongly linked to the possibility of tailoring the chelation therapy by using MRI.

## Figures and Tables

**Table 1:** Characteristics of patients undergoing a T2* MRI scan within the E-MIOT Network during the study periods.

Characteristic	Year 2019 (N = 656)	Year 2020 (N = 350)	*p*-Value
***Females, N (%)***	353 (53.8)	184 (52.6)	0.707
***Mean age (years)***	40.6 ± 11.9	39.3 ± 12.1	0.104
***Main disease, N (%)***			
***sickle cell anemia***	7 (1.1)	5 (1.4)	0.448
***sickle beta thalassemia***	15 (2.3)	8 (2.3)	
***thalassemia major***	521 (79.4)	290 (82.9)	
***thalassemia intermedia***	113 (17.2)	47 (13.4)	
***MRI scan, N (%)***			<0.0001
***first***	184 (28.0)	52 (14.9)	
***follow-up***	472 (72.0)	298 (85.1)	
***MRI LIC (mg/d dw)***	5.68 ± 7.14	6.40 ± 10.54	0.326
Global pancreas T2* (ms)	15.16 ± 11.14	14.87 ± 10.58	0.907
Global heart T2* (ms)	37.81 ± 8.06	37.57 ± 8.50	0.931

N = number, MRI = magnetic resonance imaging; LIC = liver iron concentration.

**Table 2:** Cumulative number of patients examined at each MRI center of the E-MIOT Network during the study periods.

MRI Site	Period	Number of MRI Scans in 2019	Number of MRI Scans in 2020
***Campobasso***	Entire year	56	40
	January–February	9	6
	March–June	25	6
	July–September	8	12
	October–December	14	16
***Catania***	Entire year	74	52
	January–February	17	21
	March–June	16	3
	July–September	18	23
	October–December	23	5
***Ferrara***	Entire year	202	124
	January–February	42	31
	March–June	69	17
	July–September	43	43
	October–December	48	33
***Lamezia Terme***	Entire year	43	41
	January–February	3	12
	March–June	17	1
	July–September	2	16
	October–December	21	12
***Pisa***	Entire year	168	62
	January–February	26	17
	March–June	69	3
	July–September	39	13
	October–December	34	29
***Roma***	Entire year	65	12
	January–February	20	12
	March–June	38	0
	July–September	0	0
	October–December	7	0
***Taranto***	Entire year	48	19
	January–February	1	5
	March–June	12	2
	July–September	14	8
	October–December	22	4

MRI = magnetic resonance imaging.

## Data Availability

All data can be provided by the corresponding author upon request.
